# Myopericarditis as a Delayed Complication of COVID-19 Infection: A Case Report

**DOI:** 10.7759/cureus.46655

**Published:** 2023-10-07

**Authors:** Marvi Moreno, Brianna Yee, Lubaba Haque, Kachon Lei

**Affiliations:** 1 Medical School, Kirk Kerkorian School of Medicine at the UNLV (University of Nevada, Las Vegas), Las Vegas, USA; 2 Internal Medicine, Kirk Kerkorian School of Medicine at the UNLV (University of Nevada, Las Vegas), Las Vegas, USA; 3 Cardiology, Kirk Kerkorian School of Medicine at the UNLV (University of Nevada, Las Vegas), Las Vegas, USA

**Keywords:** viral pericarditis, cardiac arrythmia, cardiomyopathy, covid-19, myopericarditis, chest pain

## Abstract

Pericarditis is the inflammation of the pericardial layers. Myopericarditis is diagnosed when this inflammation involves the myocardium, which is marked by elevated serum cardiac enzymes. With these two pathologies sharing overlaps in etiology, we present a case of a young patient with a recent history of COVID-19 infection who presented with pleuritic and positional chest pain with troponin I elevation and serial ECG changes attributed to myopericarditis as a post-viral sequela of severe acute respiratory syndrome coronavirus 2 (SARS‑CoV‑2) infection. This case demonstrates the importance of identifying and managing the potential cardiac complications in coronavirus disease 2019 (COVID-19) patients, regardless of age or symptom onset.

## Introduction

Pericarditis is referred to as the inflammation of the layers of the pericardium [1]. Based on current guidelines, a diagnosis is made based on the presence of at least two of the following criteria: chest pain, pericardial friction rub, characteristic changes in ECG, and new or worsening pericardial effusion [1]. Once the diagnostic criteria for acute pericarditis have been satisfied, a diagnosis of myopericarditis can be made when evidence of serum cardiac enzymes (creatine kinase-myocardial band (CK-MB) or troponin I or T) elevation is observed [2]. Current evidence has not demonstrated that cardiac enzyme elevation correlates with worse prognostic outcomes in patients with preserved ejection fraction, and resolution is usually observed within six months after onset [3]. Typical ECG evolution is observed in most patients, including diffuse ST elevations, followed by ST and PR normalization, T-wave inversions, and eventual ECG normalization [4]. Additionally, nonspecific echocardiographic findings include increased pericardial brightness with minimal associated effusion [2,5].

Pericarditis and myopericarditis overlap in etiology, with cardiotropic viruses being one of the primary inciting causes [2,6]. The exact mechanism is currently not well-established, but it has been proposed that severe acute respiratory syndrome coronavirus 2 (SARS‑CoV‑2) initiates cardiac damage through angiotensin-converting enzyme-2 (ACE-2) exploitation and induction of exaggerated systemic inflammation [7]. The effects of these processes may persist even during the recovery and resolution of an active infection and are hypothesized to be the cause of the delayed onset of myopericarditis presentation after the disease [2,5]. This is further supported by negative SARS-CoV-2 viral genome polymerase chain reaction (PCR) studies on autopsy examinations of nine coronavirus disease 2019 (COVID-19) patients who died from myopericarditis-associated cardiogenic shock [8].

We present a case of a young patient diagnosed with myopericarditis after the resolution of a recently confirmed COVID-19 infection.

## Case presentation

History of presentation

A 30-year-old male presented with chest pain for one day at rest. He described the pain as a squeezing sensation, exacerbated with breathing and by leaning forward. The pain interrupted his sleep, as he usually sleeps prone. The patient stated he tried aspirin 81 mg with no relief. Notably, the patient contracted COVID-19 three weeks prior with mild symptoms, confirmed positive via over-the-counter antigen testing. At that time, he used over-the-counter medications for symptom management without requiring subsequent hospitalization. On presentation to the internal medicine team, the patient was afebrile and normotensive. Heart and respiratory rates were within normal limits, and the situation of peripheral oxygen (SpO2) was >95% in room air. His physical exam was significant for pleuritic and positional chest pain. The rest of his cardiac exam was otherwise normal, without murmurs, rubs, gallops, or reproducible chest pain on palpation. Lungs were clear to auscultation bilaterally with no crackles or wheezing. Troponin I level was elevated to 241.13 pg/L (reference range: <36 pg/L) and a normal B-type natriuretic peptide (BNP) of 68 pg/mL (reference range: <100 pg/mL) [3,9]. An initial x-ray of the chest revealed no significant findings. The ECG showed no acute changes. 

Past medical history

In addition to the recent COVID-19 infection, this patient’s medical history includes anxiety and post-traumatic stress disorder. He received two doses of Pfizer-BioNTech vaccines months before his most recent infection, although he was unable to provide the exact date. Also, the patient was diagnosed with asthma after a burn pit exposure one year before this admission. The patient stated that he used albuterol before exercise. He had been prescribed amlodipine for bronchodilator-induced hypertension, though he remained normotensive without daily use. He denied any prior surgical history, and his family history was unremarkable. 

Differential diagnosis

With primary pertinent presenting information of chest pain, elevated troponin level, and this patient’s history, the differential diagnosis included inflammatory cardiac conditions (endocarditis, myocarditis, pericarditis, myopericarditis, and infiltrative heart disease), acute coronary syndrome [4,10], and acute pulmonary embolism. 

Investigations

**Figure 1:**
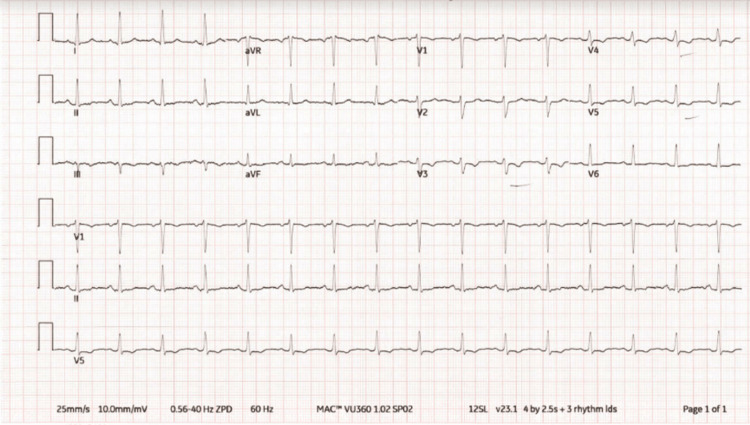
Electrocardiogram on admission.

**Figure 2:**
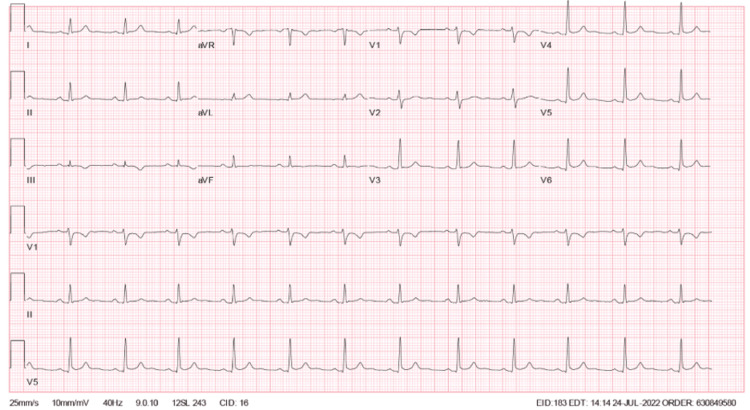
Electrocardiogram 12 hours after admission.

**Figure 3:**
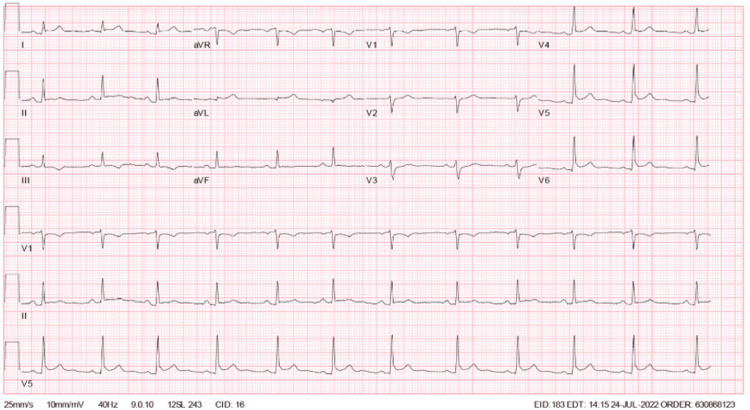
Electrocardiogram 24 hours after admission.

**Figure 4:**
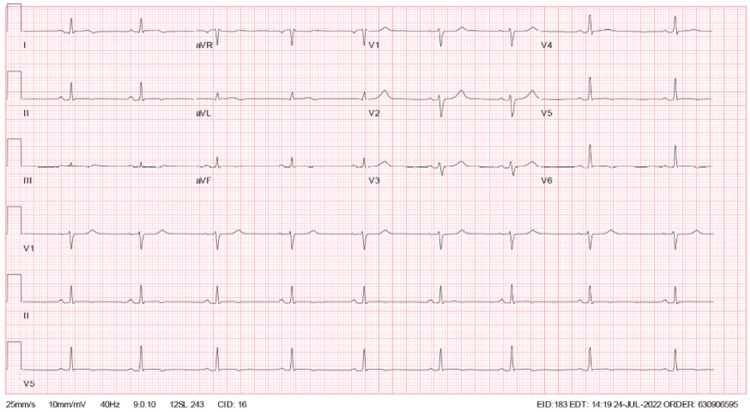
Electrocardiogram at 36 hours after admission.

The patient’s initial laboratory results were notable for within normal range complete blood count, complete metabolic panel, and erythrocyte sedimentation rate, with elevated C-reactive protein at 75. Initial troponin level was elevated at 241 pg/L, with repeat troponin levels obtained approximately every four hours. Troponin elevation peaked at the 24-hour mark from presentation to the emergency department, with a troponin level of 30,158 pg/L. Subsequent values trended down afterward. Viral PCR obtained via nasopharyngeal swab for COVID-19, influenza A and B, and respiratory syncytial virus (RSV) were negative. ECG on admission demonstrated normal sinus rhythm with nonspecific T-wave abnormalities (Figure 1). Serial ECGs were also obtained approximately every 12 hours. Repeat ECG showed ST elevations in leads V4, V5, and V6 with a concave-up appearance (Figure 2) [5]. Subsequent follow-up ECG revealed persistent findings in V4-V6 from the initial ECG, new PR depressions in leads I and II, ST depression on V1, and PR elevation in AVR (Figure 3). His last ECG before discharge showed sinus bradycardia with the resolution of abnormal ST changes (Figure 4). Chest x-ray showed no evidence of pneumonia or pleural effusion, and chest computed tomography (CT) showed slight areas of atelectasis and pulmonary artery enlargement, negative for pulmonary embolism. On admission, a transthoracic echocardiogram (TTE) showed a left ventricular ejection fraction of 50-55% with biventricular contractility in the low-normal range. Diastolic function appeared normal. Trivial, circumferential pericardial effusion was noted without tamponade. 

Management

The patient received ketorolac 15 mg intravenously and aspirin 325 mg in the emergency department with mild improvement of symptoms. He was started on colchicine 0.6 mg twice daily and ibuprofen 600 mg three times daily once admitted with the presumptive diagnosis of myopericarditis. Cardiac magnetic resonance imaging (MRI) was not available at our institution; therefore, diagnosis was made by echocardiography on the day of admission. The patient was continued on colchicine 0.6 mg once daily and ibuprofen 600 mg three times daily on subsequent days, with oxycodone 10 mg as needed for severe pain. The patient reported improved chest pain on hospital day 2, requiring fewer opioid medications for pain management. The patient was discharged on hospital day 3 with continued improvement of chest pain and was advised to taper his ibuprofen weekly after discharge with close follow-up with cardiology. He was advised to continue colchicine 0.6 mg at the same dose on discharge for three months and was counseled to abstain from strenuous activity until outpatient follow-up with cardiology.

Follow-up

The patient followed up with his primary care physician one month after discharge. The patient reported the resolution of his chest pain and had completed his ibuprofen taper before his appointment. At that time, he noted mild shortness of breath on exertion, though he had improved overall. Due to the long waitlist for a cardiology appointment, the cardiologist saw the patient 2.5 months later. The patient reported complete resolution of symptoms at that time.

## Discussion

This patient was diagnosed with myopericarditis given the presence of pleuritic and positional chest pain, non-focal and evolving ECG changes, elevated troponin levels, and preserved ejection fraction with minimal pericardial effusion demonstrated on echocardiography, likely secondary to his previous COVID-19 infection. This patient’s clinical course appeared to be mild overall. 

Diagnosis of pericarditis and myopericarditis in patients with active or recent COVID-19 is the same as in other populations, but careful attention must be observed to prevent transmission of the infection [11]. The TTE is typically the initial imaging modality used for cardiac evaluation, which was performed in this patient [12]. Findings from this can be non-specific, so cautious interpretation is warranted. 

Although the exact prevalence of cardiovascular involvement in COVID-19 patients remains unknown, a recent study has demonstrated the increased risk of cardiac injury in unvaccinated individuals [13]. Although some risk of cardiac insult remains after vaccination, this is significantly reduced, suggesting some cardioprotective effects of the vaccines from SARS-CoV-2 infection [13], which may have been attributed to this patient’s mild disease course. 

A systematic review performed by Sawalha et al. identified 14 cases, primarily males with a median age of 50.4 years, of myocarditis/myopericarditis suspected to be associated with COVID-19 infection, with 50% noted to have no comorbid conditions [14]. In contrast, a review by Kariyanna et al. included nine cases with equally affected males and females and a median age of 52 [15]. All patients reported from the included case reports demonstrated signs and symptoms during active COVID-19 infection. In contrast, we highlight a patient who presented significantly younger than those reported in previous literature, who was additionally diagnosed three weeks after the initial diagnosis of COVID-19. Delayed onset myopericarditis after the resolution of COVID-19 infection appears to be a unique feature of this patient’s presentation, suggested by the negative COVID-19 result on admission. 

Cases of myopericarditis associated with mRNA COVID-19 vaccines have been reported, though the presentation usually occurs within days of immunization [16,17]. On the other hand, studies more commonly noted the incidence of inflammatory cardiac conditions occurring within weeks to months after contracting the infection [18,19]. Given the temporal relationship between his COVID-19 infection and the presentation of chest pain, his observed symptoms were attributed to his recent COVID-19 infection, as no other obvious causal relationship could be found. 

Treatment of myopericarditis warrants a higher level of evidence due to the limited clinical trials available [16]. A systematic review conducted by Imazio et al. showed that the most common treatment for idiopathic and viral pericarditis in European countries and Northern America is non-steroidal anti-inflammatory drugs (NSAIDs), with colchicine as an adjunct of choice, reducing recurrence by 50% [20]. For those who fail to respond, corticosteroids are suggested to be the second-line in medical management [21]. Our patient was initially managed with ibuprofen and colchicine with a positive response. The current literature needs more data on the efficacy of this management strategy on COVID-19-induced myopericarditis.

## Conclusions

Findings of COVID-19-associated myopericarditis appear similar to those caused by more well-known etiologies. Although COVID-19-induced myopericarditis typically presents in older individuals with active disease, we highlight the importance of considering myopericarditis in young patients with a recent history of SARS-CoV-2 infection presenting with chest pain, cardiac enzyme elevation, and ECG changes. The lack of data regarding COVID-19-induced myopericarditis pathophysiology, diagnostic protocol, and medical management warrants further research.

## References

[1] Welch T (2023). Management of acute and recurrent pericarditis. J Am Coll Cardiol.

[2] Imazio M, Cecchi E, Demichelis B (2008). Myopericarditis versus viral or idiopathic acute pericarditis. Heart.

[3] Novack ML, Zevitz ME (2023). Natriuretic peptide B type test. StatPearls [Internet].

[4] Smulders MW, Kietselaer BL, Schalla S (2016). Acute chest pain in the high-sensitivity cardiac troponin era: a changing role for noninvasive imaging?. Am Heart J.

[5] Imazio M, Cooper LT (2013). Management of myopericarditis. Expert Rev Cardiovasc Ther.

[6] Imazio M, Trinchero R (2008). Myopericarditis: etiology, management, and prognosis. Int J Cardiol.

[7] Akhmerov A, Marbán E (2020). COVID-19 and the heart. Circ Res.

[8] Del Nonno F, Frustaci A, Verardo R (2022). Virus-negative myopericarditis in human coronavirus infection: report from an autopsy series. Circ Heart Fail.

[9] Samman Tahhan A, Sandesara P, Hayek SS (2023). High-sensitivity troponin I levels and coronary artery disease severity, progression, and long-term outcomes. J Am Heart Assoc.

[10] (2022). Causes of Non ACS Related Troponin Elevations. https://www.acc.org/Latest-in-Cardiology/Articles/2014/07/18/13/16/Causes-of-Non-ACS-Related-Troponin-Elevations.

[11] Patone M, Mei XW, Handunnetthi L (2022). Risk of myocarditis after sequential doses of COVID-19 vaccine and SARS-CoV-2 infection by age and sex. Circulation.

[12] Mele D, Flamigni F, Rapezzi C, Ferrari R (2021). Myocarditis in COVID-19 patients: current problems. Intern Emerg Med.

[13] D'Andrea A, Di Giannuario G, Marrazzo G (2020). The role of multimodality imaging in COVID-19 patients: from diagnosis to clinical monitoring and prognosis [Article in Italian]. G Ital Cardiol (Rome).

[14] Sawalha K, Abozenah M, Kadado AJ (2021). Systematic review of COVID-19 related myocarditis: insights on management and outcome. Cardiovasc Revasc Med.

[15] Kariyanna PT, Sutarjono B, Grewal E (2020). A systematic review of COVID-19 and myocarditis. Am J Med Case Rep.

[16] Patone M, Mei XW, Handunnetthi L (2022). Risks of myocarditis, pericarditis, and cardiac arrhythmias associated with COVID-19 vaccination or SARS-CoV-2 infection. Nat Med.

[17] Fatima M, Ahmad Cheema H, Ahmed Khan MH (2022). Development of myocarditis and pericarditis after COVID-19 vaccination in adult population: a systematic review. Ann Med Surg (Lond).

[18] Zuin M, Rigatelli G, Bilato C, Porcari A, Merlo M, Roncon L, Sinagra G (2023). One-year risk of myocarditis after COVID-19 infection: a systematic review and meta-analysis. Can J Cardiol.

[19] Bajaj R, Sinclair HC, Patel K (2021). Delayed-onset myocarditis following COVID-19. Lancet Respir Med.

[20] Theetha Kariyanna P, Sabih A, Sutarjono B (2022). A systematic review of COVID-19 and pericarditis. Cureus.

[21] Imazio M, Gaita F, LeWinter M (2015). Evaluation and treatment of pericarditis: a systematic review. JAMA.

